# Comparative hazard analysis and toxicological modeling of diverse nanomaterials using the embryonic zebrafish (EZ) metric of toxicity

**DOI:** 10.1007/s11051-015-3051-0

**Published:** 2015-06-04

**Authors:** Bryan Harper, Dennis Thomas, Satish Chikkagoudar, Nathan Baker, Kaizhi Tang, Alejandro Heredia-Langner, Roberto Lins, Stacey Harper

**Affiliations:** Oregon State University, 1007 ALS Building, Corvallis, OR 97331 USA; Pacific Northwest National Laboratory, Richland, WA USA; Intelligent Automation, Inc., Rockville, MD USA; CPqAM, Oswaldo Cruz Foundation, FIOCRUZ-PE, Recife, PE 50.740-465 Brazil

**Keywords:** Nanoparticle, Zebrafish, Toxicity, Surface chemistry, Informatics

## Abstract

The integration of rapid assays, large datasets, informatics, and modeling can overcome current barriers in understanding nanomaterial structure–toxicity relationships by providing a weight-of-the-evidence mechanism to generate hazard rankings for nanomaterials. Here, we present the use of a rapid, low-cost assay to perform screening-level toxicity evaluations of nanomaterials in vivo. Calculated EZ Metric scores, a combined measure of morbidity and mortality in developing embryonic zebrafish, were established at realistic exposure levels and used to develop a hazard ranking of diverse nanomaterial toxicity. Hazard ranking and clustering analysis of 68 diverse nanomaterials revealed distinct patterns of toxicity related to both the core composition and outermost surface chemistry of nanomaterials. The resulting clusters guided the development of a surface chemistry-based model of gold nanoparticle toxicity. Our findings suggest that risk assessments based on the size and core composition of nanomaterials alone may be wholly inappropriate, especially when considering complex engineered nanomaterials. Research should continue to focus on methodologies for determining nanomaterial hazard based on multiple sub-lethal responses following realistic, low-dose exposures, thus increasing the availability of quantitative measures of nanomaterial hazard to support the development of nanoparticle structure–activity relationships.

**Electronic supplementary material:**

The online version of this article (doi:10.1007/s11051-015-3051-0) contains supplementary material, which is available to authorized users.

## Introduction

Scientists and engineers, whether in industry, government, or academia, have a common need to understand how nanomaterials interact with biological systems to mitigate potential risks and to define structure–activity relationships (SARs) that can be used to predict nanomaterial fate and hazard in lieu of empirical data (Fourches et al. 2010; Hristozov et al. 2014; Rallo et al. 2011; Zhang et al. 2012). In consideration of nanomaterial complexity, ‘structure’ needs to be descriptive of inherent nanomaterial features such as the number of atoms, surface charge, crystallinity, surface area, etc.; whereas, ‘activity’ would represent the conditional behaviors of those nanomaterials such as zeta potential, biological activity, and size distribution, among others (Sayes 2014). Engineered nanomaterials pose a tremendous analytical challenge as they encompass all we know about the complexities of biochemistry, coupled with new characteristics associated with their biologically relevant size and increased relative surface area for interacting with biological systems (Gajewicz et al. 2012). Furthermore, simple changes in the nanoparticle (NP) exposure media can significantly alter the uptake and toxic responses elicited by the same nanomaterial (Kim et al. 2013; Truong et al. 2011). As a result, developing predictive methods of understanding engineered NP risks requires analysis of a range of NP compositions and conditions, thus demanding rapid, cost-effective testing strategies that can keep pace with innovation (Nel et al. 2012; Vecchio et al. 2014).

**Fig. 1:**
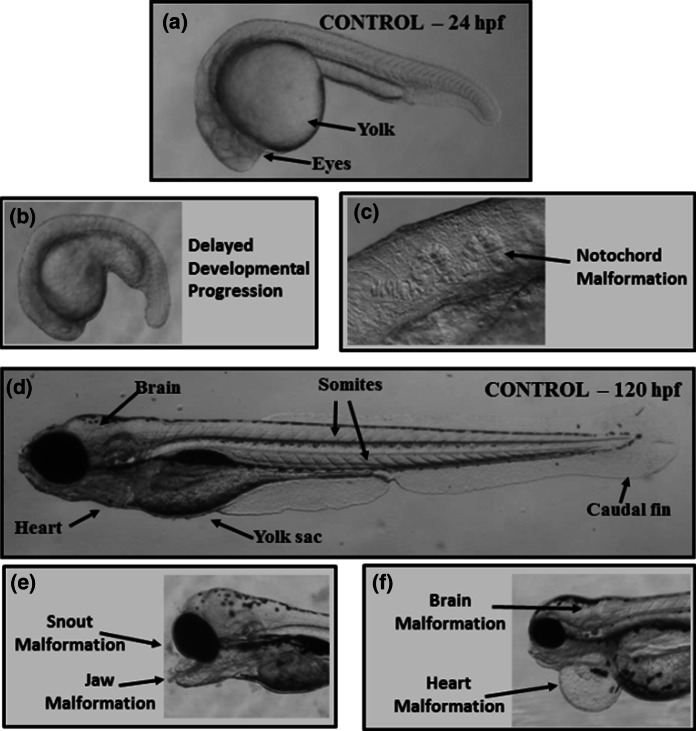
Overview of morphological endpoints assessed during the EZ Metric assay including **a** image of control zebrafish embryo at 24 h post fertilization (hpf), **b** image of zebrafish embryo exhibiting delayed developmental progression, **c** image of wavy notochord malformation in 24-hpf zebrafish embryo, **d** image of control zebrafish at 120 hpf, **e** image of snout and jaw malformations observed in 120-hpf zebrafish, and **f** image of brain and heart malformations (pericardial edema) in 120-hpf zebrafish

Current toxicological methods are costly and time consuming, not always applicable to nanomaterials in suspension, and often require large quantities of materials (Rushton et al. 2010). These methods often struggle not only with understanding appropriate dose metrics for nanomaterials, but too often rely on costly LC_50_ data in the absence of a thorough understanding of low-dose, sub-lethal effects (Maynard et al. 2011; Oberdörster 2010). Novel toxicological methods need to look at realistic exposure levels during first-pass hazard identification studies to minimize the time and materials required for testing and rapidly identify materials of high concern (Oomen et al. 2014). The EZ Metric assay presented here utilizes developing zebrafish embryos (*Danio rerio*) as an integrated sensing and amplification system that is easy to evaluate non-invasively, providing the power of whole-animal investigations with the convenience of cell culture (Harper et al. 2008a; Usenko et al. 2007). Exposures are conducted in 96-well plates using intact organisms that have functional homeostatic feedback mechanisms and intercellular signaling (Harper et al. 2010, 2011; Truong et al. 2011).The endpoints evaluated in the EZ Metric assay require minimal equipment to assess and involve no experimental treatments such as dyes or other indicators that could alter the impacts of the nanomaterials (Harper et al. 2008a; Truong et al. 2011). All endpoints are observed under low-power magnification using dissecting scopes (Fig. 1), methods that lend themselves to potential automated visual analyses (Hans et al. 2013). Given these unique advantages, embryonic zebrafish are becoming widely used to screen chemicals and nanomaterials through automated robotic testing platforms coupled with various types of automated optical analysis (Mandrell et al. 2012; Nel et al. 2012; Truong and Reif 2014; Truong et al. 2012).

In order to consider multiple endpoints measured in the zebrafish as an integrated measure of toxicity valuable for developing predictive models, we assessed individual endpoints and weighted those responses relative to their theoretical biological impacts. The weighted responses were used to calculate an EZ Metric score representative of the integrated biological response at each exposure concentration. Data are available through the Nanomaterial-Biological Interactions (NBI) knowledgebase (nbi.oregonstate.edu). Median effect levels (EC_50_) estimated from the weighted EZ Metric scores of 68 different nanomaterials were used to determine a hazard ranking. We were interested in testing the hypothesis that groups of NPs with similar toxicity have similar chemical/structural characteristics such as size, surface chemistry, or core composition. Conversely, NPs that are not functionally similar should have varying toxicity. Thus, the identification of NPs with similar EZ Metric-concentration profiles should be indicative of NP features that serve as drivers of nanomaterial–biological interactions leading to toxicity.

The goals of this study were (1) to provide a hazard ranking of the diverse nanomaterials housed on the NBI Knowledgebase, (2) to identify inherent characteristics of nanomaterials useful in the development of predictive models of toxicity, and (3) to highlight the importance of realistic dosing scenarios and analysis of sub-lethal effects in the development of models designed to identify NP hazard. Herein, we present data supporting both the utility of the assay in achieving the above-stated goals and a surface chemistry-based model of NP toxicity to developing zebrafish.

## Methods

### Nanomaterials

Nanomaterials were acquired from a variety of commercial sources including Sigma Aldrich (St. Louis, MO, USA), Nanocomposix (San Diego, CA, USA), Dendritic Technologies (San Francisco, CA USA), and non-commercial research labs. Details of nanomaterial manufacturers and material composition are available in Online Resource 1, online at nbi.oregonstate.edu and in previous publications on selected materials (Harper et al. 2007, 2008a; Pryor et al. 2014; Usenko et al. 2007, 2008).

### Embryonic zebrafish care and preparation

Zebrafish (*Danio rerio*) embryos were collected from group spawns of wild-type D5 fish housed at the Sinnhuber Aquatic Research Laboratory (Oregon State University, Corvallis Oregon). The chorion surrounding the embryo was removed enzymatically at 6 h post fertilization (hpf) (Usenko et al. 2007) to ensure that nanomaterials were in contact with the developing embryos. Dechorionation was performed by exposing groups of 200–400 embryos to 1.5 ml of 50 mg/ml protease from *Streptomyces griseus* (Sigma Aldrich cat#81750) in a 60-mm glass petri dish for approximately 6 min until the chorions begin to detach, then gently rinsing the embryos several times with fishwater to complete the removal. Fishwater was prepared by diluting 0.26 g/L Instant Ocean salts (Aquatic Ecosystems, Apopka, FL) into reverse osmosis (RO) water and adjusting the pH to 7.2 ± 0.2 with sodium bicarbonate. Embryos at 8 hpf were exposed in clear, 96-well exposure plates, one animal per well, to various concentrations of exposure solutions as previously described by Harper and colleagues (2008b).

### EZ Metric assay

Exposures’ concentrations were typically fivefold serial dilutions of nanomaterials ranging from approximately 250 parts per million (ppm) down to ~16 parts per billion (ppb) prepared in fishwater. Control exposures comprised fishwater alone (without NPs). Embryos were incubated at 26 °C under 14/10 light cycle and were evaluated visually at 24 hpf for viability, developmental progression, and spontaneous movement (earliest behavior in zebrafish). At 120 hpf, behavioral endpoints (motility, tactile response) were thoroughly evaluated in vivo and larval morphology (body axis, eye, snout, jaw, otic vesicle, notochord, heart, brain, somite, fin, yolk sac, trunk, circulation, pigment, swim bladder) was evaluated visually and scored in a binary fashion (present or absent) (Harper et al. 2008a, b; Truong et al. 2011).

### Weighted EZ Metric score

To summarize the 21 measured toxicity endpoints for each dose applied to the embryonic zebrafish, we define the EZ Metric score to provide a relative comparison of nanomaterial-elicited effects. In a previous publication, we compared the predictive ability of an additive approach to summarizing the 21 endpoints and found that weighting the biological impacts of each individual endpoint provided better predictability of nanomaterial toxicity (Liu et al. 2013). As such, for this analysis, a weighted EZ Metric score was calculated for each exposure concentration by multiplying the frequency of an individual endpoint occurrence at a given concentration by the endpoint weighting factor found in Table 1 and normalizing for the number of viable embryos displaying that effect. Weighting factors were based on consensus ranking of the severity of each sub-lethal endpoint, such that embryos surviving until 120 hpf with developmental abnormalities have scores reflecting the severity of those combined sub-lethal effects as follows:

1 $${\text{Weighted}}\;{\text{EZ}}\;{\text{Metric}} = \sum\limits_{i = 0}^{n} {w_{i} \times (E_{i} )} ,$$

where *w*_*i*_ is the weight from Table 1 for the *i*th biological effect E_*i*_.

**Table 1:** Ranking of endpoints assessed in zebrafish embryos and their associated weighting used for calculation of the overall EZ Metric score

EZ Metric Endpoint	Weighting factor
24 hpf mortality	1.0
120 hpf mortality	0.95
Heart malformation	0.12
Brain malformation	0.12
Yolk sac edema	0.1
Notochord malformation	0.08
Curved axis	0.08
Trunk malformation	0.06
Delayed developmental progression	0.06
Occluded circulation	0.04
Eye malformation	0.04
Jaw malformation	0.04
Lack of spontaneous movement	0.04
Somite malformation	0.02
Motility	0.02
Lack of touch response	0.02
Snout malformation	0.02
Otic malformation	0.02
Caudal/pectoral fin malformation	0.02
Atypical pigmentation	0.02
Atypical swim bladder inflation	0.02

EZ Metric data are made publically available through the Nanomaterial-Biological Interactions knowledgebase at http://nbi.oregonstate.edu

### Statistical analysis and modeling

Spearman rank correlations were conducted using SigmaPlot Version 12.0 (Systat Software Inc.). Estimation of the median effect level (EC_50_) based on EZ Metric score was achieved through linear interpolation as many nanomaterials tested did not elicit significant toxicological effects at the highest concentrations tested, precluding an accurate determination of the EC_50_ values through traditional logistic or sigmoidal regression models. MATLAB hierarchical clustering algorithm with Euclidean distance measure and Ward linkage rule was used to identify groups of nanoparticles that were functionally similar with respect to their EZ Metric values across the entire concentration range. Following clustering analysis, classification analysis was performed using the open source Weka software (v. 3.6.3) implementing the C4.5 decision tree algorithm with tenfold cross-validation. Supervised learning of identified target classes was used to identify the features common to the samples in each cluster. In order to compare the distribution of EZ Metric values across exposure concentrations within the identified clusters, we defined a quantity called sumEZ, such that,

2 $${\text{sumEZ}} = \sum\limits_{i = 1}^{8} {{\text{EZmetric}}_{i} } ,$$

where EZMetric_*i*_ is the EZ Metric value at the *i*th concentration of the nanoparticle.

For the surface chemistry-based toxicological model of NP toxicity, a subset of gold nanoparticles with differing surface chemistries and otherwise similar structures were isolated and the surface functional group chemical characteristics were built using the extensible computational chemistry environmental program (Black et al. 2003). The compounds were geometry optimized at the Hartree–Fock/6-31G* level of theory using the NWChem 5.1 program (Bylaska et al. 2007; Kendall et al. 2000) and the band gaps calculated. The remaining topographical and physicochemical molecular descriptors were calculated using the Cerius2/Discovery Studio program (Accelrys 2006). Physicochemical parameter estimates and chemical attributes used in model development are provided in Table 2.

**Table 2:** Values for the molecular descriptor variables for each surface modification used in model development

Variable, units	MEE	MEEE	TMAT	MES
SASA, Å^2^	344.15	438.46	286.83	314.97
SASA/Polar^a^	5.04	5.66	7.39	3.02
Refractivity (m^3^/mol)	31.78	42.82	48.38	28.01
Band Gap (kcal/mol)	−211.8	−211.7	−215.8	−195.3

Polar Surface (Å2) is the surface area formed by all the polar atoms of a molecule, Solvent-Accessible Surface Area (SASA, Å2) is the surface area of a molecule available to a spherical solvent molecule, Molar Refractivity (Refractivity, m3/mol) is a measure of the volume occupied by an atom or functional group, and Band Gap (kcal/mol) is the energy difference between the highest occupied molecular orbital (HOMO) and the lowest occupied molecular orbital (LUMO)

^a^Unitless quantity

## Results

### Nanoparticle toxicity

**Fig. 2:**
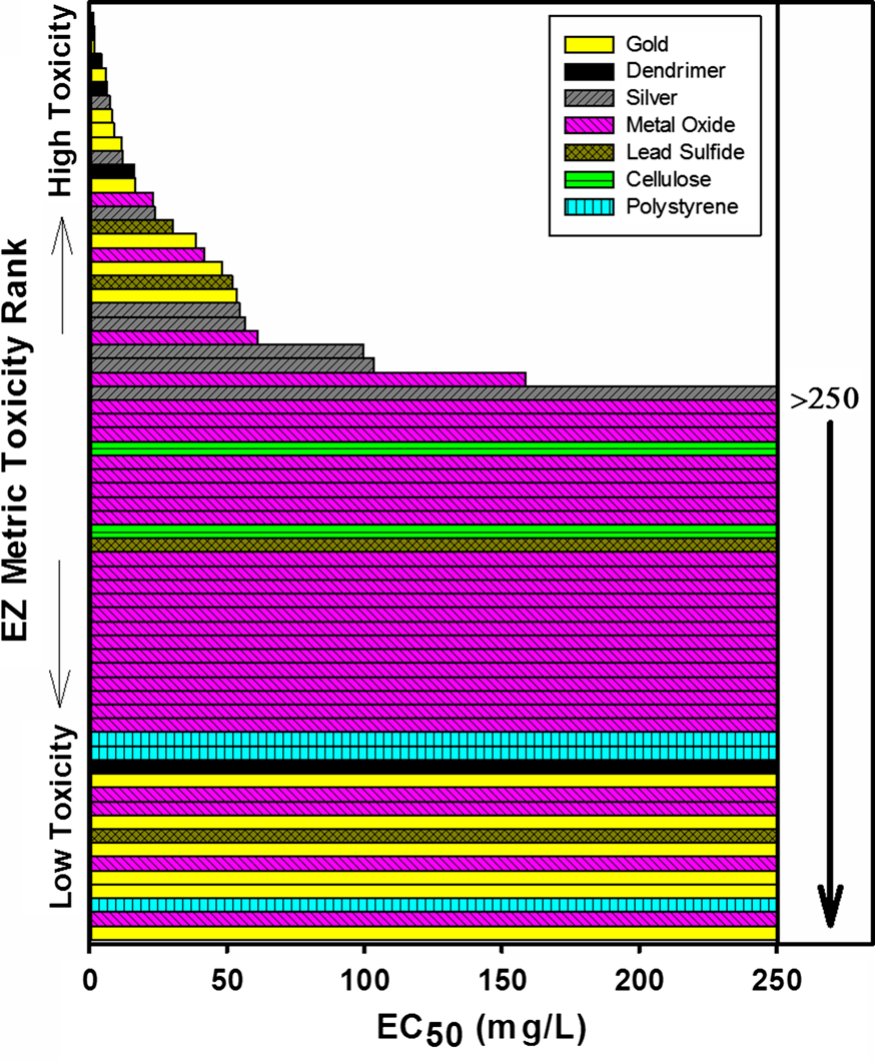
Hazard ranking of nanomaterials based on EC_50_ dose for EZ Metric score

To compare the toxicity of the diverse nanomaterials, weighted EZ Metric values were plotted against log-transformed nanoparticle exposure concentrations to estimate the median effect level (EZ Metric Score = 0.5 or EC_50_). Hazard ranking based on weighted EZ Metric EC_50_ values is shown in Fig. 2, and NP descriptors associated with each value are detailed in Table 3. For nanomaterials where the toxicity did not result in a 50 % effect in EZ Metric score at the highest dose tested (~250 ppm), we ranked those materials based on the exposure concentration resulting in a weighted EZ Metric score equal to 0.1 (values listed in Online Resource 2).

**Table 3:** Median weighted EZ Metric exposure concentrations (EC_50_) determined in embryonic zebrafish following 5-day exposure to the various types of nanoparticles

Material	EC_50_
Gold-TMAT (2 nm)-as synthesized	0.2
Gold-TMAT (0.8 nm)	1.3
G3 PAMAM dendrimer—amine	1.7
Gold-TMAT (2 nm)-pure	1.9
G5 PAMAM dendrimer—amine	4.3
Gold—phosphatidylcholine (14 nm)	6.2
G4 PAMAM dendrimer—amine	6.2
Silver—citrate (10 nm)	7.4
Gold-TMAT (2 nm)-ultrapure	8.1
Gold—phosphatidylcholine (14 nm)	9.0
Gold—phosphatidylcholine (22 nm)	11.6
Silver/Gold—phosphate (68 nm)	12.2
G6 PAMAM dendrimer—amine	16.5
Gold-TMAT (2 nm)-ultrapure	16.7
Erbium Oxide (25 nm)	23.2
Silver/Gold—phosphate (92 nm)	23.7
Lead Sulfide—monothiol, oxidized (3 nm)	30.4
Gold—phosphatidylcholine (7 nm)	38.9
Samarium Oxide (25 nm)	41.7
Gold-MHA (10 nm)	48.2
Lead Sulfide—monothiol, unoxidized (3 nm)	51.9
Gold—phosphatidylcholine (7 nm)	53.7
Silver/Gold—phosphate (61 nm)	54.6
Silver/Gold—phosphate (70 nm)	56.6
Holmium oxide (25 nm)	61.3
Silver/Gold—phosphate (101 nm)	99.7
Silver/Gold—phosphate (122 nm)	103.5
Dysprosium oxide (25 nm)	158.5

Nanoparticles are listed from most to least toxic as is represented in Fig. 2

Analysis of the hazard ranking shown in Fig. 2 reveals distinct patterns of toxicity related to the outermost surface chemistry of the nanomaterials. Four of the seven core compositions spanned the range of observed toxicity depending on their surface chemistry (Fig. 2). Both dendrimer and gold samples show dramatic differences in toxicity associated with changes in the surface chemistry of otherwise similar particles. Positively charged amine-functionalized dendrimers and *N*,*N*,*N*-trimethylammoniumethanethiol (TMAT)-functionalized gold nanoparticles were significantly more toxic than their neutral or negatively charged counterparts with other surface chemistries. Pure metal oxide nanoparticles were found to vary in their overall hazard ranking with core composition; however, surface chemical modifications did affect metal oxide NP toxicity as illustrated by the ranking of a series of zinc oxide nanoparticles with varying surface chemistries and similar core sizes and composition (Online Resource 2). Spearman rank correlation analysis of NP core composition and outermost surface chemistry showed that both factors have significant correlation with EC_50_ values, with surface chemistry having a higher correlation coefficient (0.75, *p* < 0.001, *n* = 28) than core composition (0.54, *p* = 0.003, *n* = 28).

### Clustering analysis

**Fig. 3:**
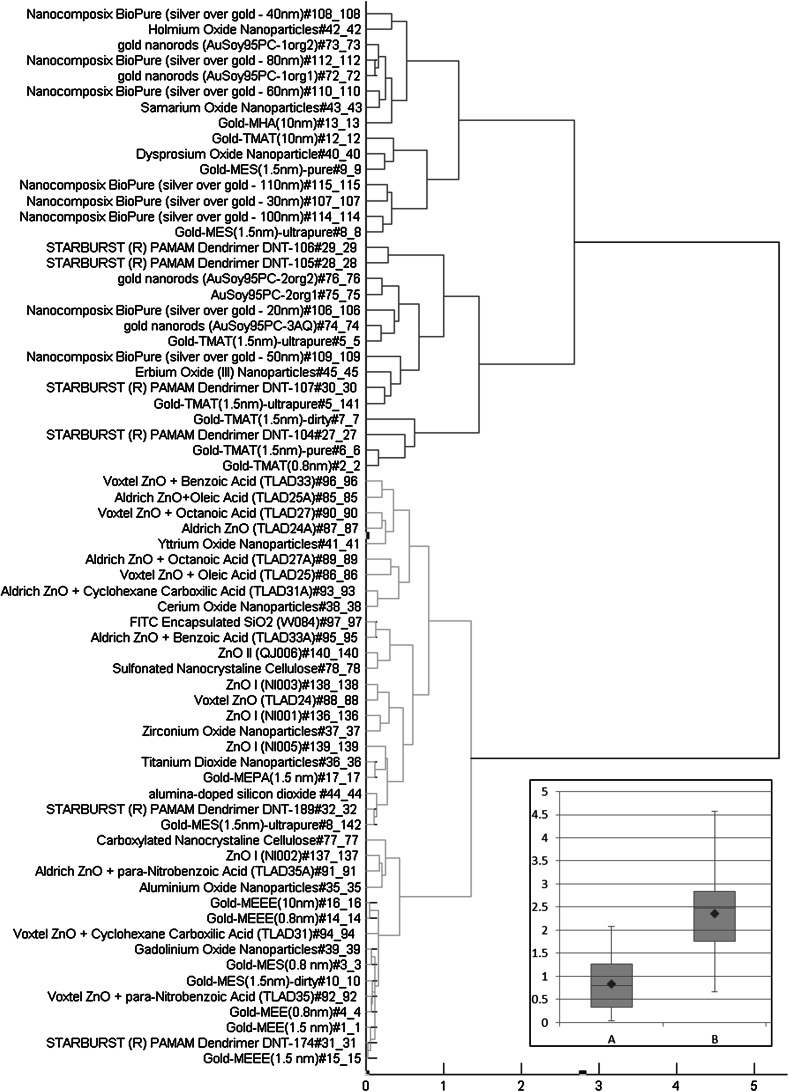
Dendrogram plot showing the hierarchical clustering of 68 nanoparticle samples based on their weighted EZ Metric scores. Clustering analysis is done using MATLAB. The clustering method uses the Ward linkage rule with Euclidean distance measure. Clusters A (*Blue*) and B (*Red*) are the top-level clusters identified in the plot. Insert—Box plots of sumEZ values for clusters A and B. The *red-colored solid diamond* symbol represents the mean of sumEZ values in each cluster. (Color figure online)

Cluster analysis was performed on the overall dataset to identify inherent nanoparticle features that serve as good predictors of nanoparticle toxicity. Nanoparticles can be grouped into different clusters based on similarity/dissimilarity in their EZ Metric-concentration profile. Hierarchical clustering methods based on Ward linkage rule with Euclidian distance measure gave well-separated clusters for EZ Metric scores when data were assessed on the chemical constituents, primary particle size, and surface chemistry of the nanomaterials (Fig. 3). Other linkage rules (e.g., single linkage) and distance measures (e.g., Manhattan distance measure) did not yield clusters that were as well separated from each other (data not shown). Clustering analysis of these nanomaterials based on the weighted EZ Metric score revealed two clusters, indicating that the outermost surface chemistry of the nanomaterial was a stronger predictor of toxicity than any other independent feature (Fig. 3). A rough comparison of the distribution of EZ Metric values of cluster A with that of cluster B was conducted using a consolidation estimate, sumEZ (Eq. 2). This analysis revealed that the toxicity of cluster B nanoparticles was higher than that of cluster A based on their respective weighted EZ Metric scores (Fig. 3, insert).

**Fig. 4:**
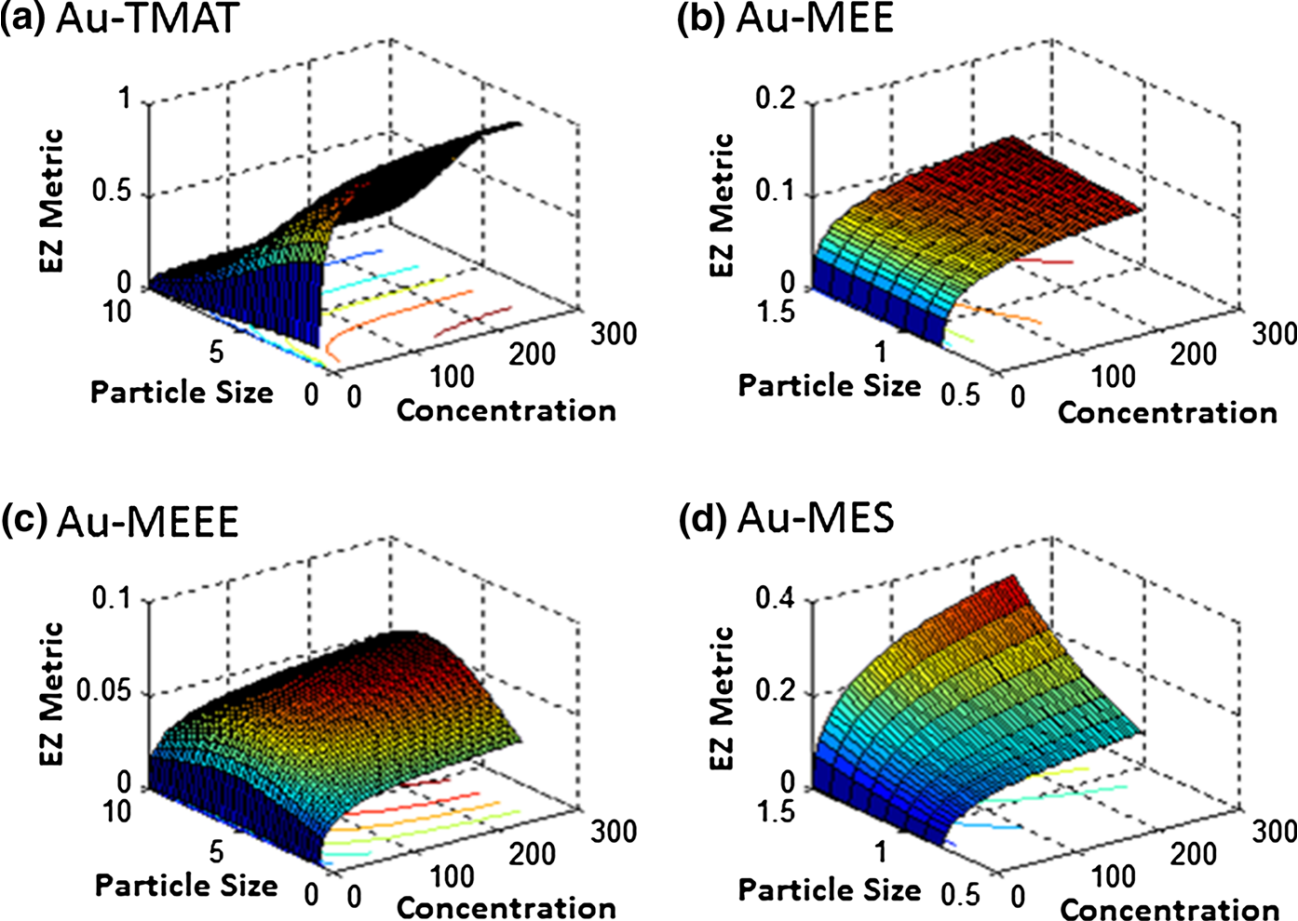
Prediction surface plots of EZ Metric values (*z*-axes) obtained with the model in Eq. 3 as a function of particle size (*x*-axes) and concentration (*y*-axes). Results for gold nanoparticles with **a** TMAT, **b** MEE, **c** MEEE, and **d** MES surface ligands are shown on the *top left*, *top right*, *bottom left*, and *bottom right* panels, respectively

Identifying nanoparticle features distinguishing cluster A nanoparticles from cluster B nanoparticles was performed to identify those features useful for predicting nanoparticle hazard. To this end, we constructed a decision tree model based on nanoparticle surface components using the WEKA J48 algorithm. As shown in the dendrogram in Fig. 4, nanoparticles were grouped into clusters A and B based on the presence/absence of four surface functional groups, namely TMAT, phosphate, phosphatidylcholine, and amine. Based on these four features with leave-one-out cross-validation, the J48 classifier was able to classify the nanoparticles into the two clusters with 94 % accuracy. Four nanoparticles were misclassified using this approach: gold-TMAT (10 nm) and Nanocomposix BioPure (silver over gold −30 nm) were misclassified into cluster A’; gold-MES (1.5 nm)-ultrapure and erbium oxide (III) nanoparticles were misclassified into cluster B’.

Group analysis of nanoparticles that are functionally similar with respect to toxicity showed that the membership of each group can differ slightly depending on which of the 21 individual endpoint variables is used as the characteristic measure of nanoparticle toxicity; however, in all cases, clustering analysis defined two groups of nanoparticles. The analysis further revealed that nanoparticles that have any of the four surface components including TMAT, phosphate, phosphatidylcholine, and amine are mostly similar with respect to their toxicity, irrespective of which characteristic of the EZ Metric was used for clustering. Some minor misclassifications based on the presence/absence of these surface components were identified and attributed to variation in other features (e.g., size or concentration of surface components) that were not included in the current analysis.

### Toxicological response modeling

Clusters identified in this work were used as a basis for identifying regions in predictor feature space where linear predictive models of nanoparticle toxicity could be developed (Silva et al. 2014). To investigate the relationship between weighted EZ Metric scores and the inherent nanoparticle features, we focused on a subset of four gold nanoparticles that varied in surface chemistry and size. Physicochemical properties and chemical attributes shown in Table 2 were calculated from atomic models and used as independent variables, along with the natural logarithm of concentration and primary particle size, to build a model for predicting the EZ Metric response. The logarithmic transformation of the values of an experimental factor like concentration is useful when the variable has a large range (Finney 1947; Myers et al. 2001). Thus, original EZ Metric responses were transformed by multiplying the score by 100 and adding 0.1 to avoid the discontinuity resulting from taking the natural logarithm of zero scores (denoted as Mod EZ Metric in Eq. 3). Studying the diagnostics of the model, the presence of two outliers became evident. Outliers were removed and a model was fit to the resulting dataset. The final model has the form shown in Eq. 3.

3 $$\begin{aligned} {\text{Mod}}\;{\text{EZ}}\;{\text{Metric}} = & \exp \left( {\beta_{0} + \beta_{1} \log ({\text{Conc}}) + \beta_{2} {\text{Size}} + \beta_{3} {\text{SASA/Polar}}} \right. \\ + \beta_{4} {\text{Refractivity}} + \beta_{5} {\text{Band}}\;{\text{Gap}} + \beta_{13} \log ({\text{Conc}}) \cdot {\text{SASA/Polar}} \\ \left. { + \beta_{25} {\text{Size}} \cdot {\text{Band}}\;{\text{Gap}} + \beta_{11} [\log ({\text{Conc}})]^{2} + \beta_{22} {\text{Size}}^{2} } \right), \\ \end{aligned}$$

where *β*_*i*_ are model parameters estimated using nonlinear least squares and log(Conc) represents the natural logarithm of the NP concentration used in the tests (modified as described in the previous paragraph). *Size* is the primary NP diameter in nanometers, *SASA* is the Solvent-Accessible Surface Area (Å^2^), *Polar* represents the surface area formed by all the polar atoms of a molecule (Å^2^), *Refractivity* is the molar refractivity, a measure of the volume occupied by an atom or functional group (m^3^/mol), and *Band Gap* is the energy difference between the highest occupied molecular orbital (HOMO) and the lowest occupied molecular orbital (LUMO). Model parameter estimates, *β*_*i*_, for each of the terms in Eq. 3, and their respective standard errors and p-values are shown in Table 4. The subscripts in the model parameter estimates (*β*_*i*_) do not have any particular meaning and are simply used to distinguish the parameter that corresponds to each term in the equation. Other NP inherent features that did not significantly alter toxicological predictability include the distribution coefficient (logD) and partitioning coefficient (logP) for the chemicals in octanol and water, the polarizability of the compound, and the van der Waals surface area (data not shown).

**Table 4:** Model parameter estimates and their corresponding standard errors and *p*-values

Model term	Estimate	Standard error	*p* value
Intercept	67.2828	5.4948	<0.0001
Log concentration	0.8128	0.0545	<0.0001
Size	14.7335	1.9790	<0.0001
SASA/Polar^a^	4.1535	0.2880	<0.0001
Refractivity, m^3^/mol	−0.2974	0.0367	<0.0001
Band gap, kcal/mol	0.3715	0.0279	<0.0001
LogC × (SASA/Polar)	−0.0547	0.0070	<0.0001
Size × Band Gap	0.0682	0.0093	<0.0001
(Log Conc)^2^	−0.0343	0.0039	<0.0001
Size^2^	−0.0255	0.0092	0.0057

^a^Unitless quantity

A plot of predicted against measured EZ Metric values obtained using the model shown in Eq. 3 illustrates that the model fits the observed EZ Metric responses relatively well (*R*^2^ = 0.88) and that the model is able to follow the general trend of the response, despite the large variability in the observed responses (Online Resource 3). Response surface plots of the model in Eq. 3 as a function of particle size and concentration indicate how surface functional groups dramatically alter the predicted toxicity of each of the four surface-modified gold nanoparticles (Fig. 4). Highest toxic responses were modeled for Au-TMAT NPs, especially at low concentrations with small particle sizes (Fig. 4a); meanwhile, Au-MES model results indicate an opposite response, with increasing toxicity as particle size increases (Fig. 4d). Based on the model, Au-MEE is predicted to have similar toxicity across particle sizes as responses were only affected by particle concentration (Fig. 4b), while Au-MEEE response plots suggest some role of size in toxicity despite these AuNPs being overwhelmingly low in toxicity (Fig. 4c).

## Discussion

The studies presented here show that the rapid, reliable, and cost-effective EZ Metric assay can be used to assess integrated living system responses at realistic exposure concentrations and provide the information necessary for predictive modeling and determination of hazard, all while providing insights into potential mechanisms of toxicity. Through this study, we have gained insight into the important nanomaterial features that govern their interactions with biological systems. Based on analysis across multiple material types, the assay has revealed that the outermost surface chemistry of nanomaterials is a strong determinant in their overall in vivo toxicity.

The use of large datasets and modeling approaches across a wide range of data, all collected using the same experimental methodologies, overcomes some of the previous barriers noted in the development of nanomaterial structure–toxicity relationships (Cohen et al. 2012; Rallo et al. 2011; Zhang et al. 2012). We are unaware of any previous analyses that studied this quantity of diverse nanomaterials simultaneously, using the same vertebrate assay, with the goal of identifying the inherent NP features predictive of hazard. The weighted ranking of EZ Metric assay endpoints allows for all measurable sub-lethal effects to be taken into account, an approach applicable to modeling of biological interactions at realistic exposure levels. Mechanistic hypothesis generation is often difficult following first-pass hazard identification studies that rely on mortality alone or are not conducted at realistic doses, difficulties that are overcome with our approach. The time and cost associated with the EZ Metric approach is significantly less than traditional in vivo methods, yet it provides data on numerous sub-lethal endpoints valuable for mechanistic hypothesis generation related to the mode of toxicity.

 In addition to the research-driven benefits of informatics approaches that integrate large amounts of diverse data, predictive models developed from structured datasets also inform safer design rules for nanomaterial engineering (Harper et al. 2013). When combined with informatics approaches, the EZ Metric assay and scoring technique can be used to develop safer design rules and to provide insight into structure–property relationships that exist across nanomaterials. Informatics can provide a data integration platform for consolidating the weight-of-the-evidence, thus supporting research into novel applications and at the same time informing safe design rules for nanoengineering. Incorporating toxicological evaluations early in research and development schemes will allow us to close the testing–redesign loop and favor the development of nanomaterials with minimal toxicity (Harper et al. 2008b, 2010). Given the immense need to quickly and cost-effectively screen chemicals and nanomaterials for their toxicity, data-rich assays like the EZ Metric that are (i) rapid, (ii) readily amenable to inter-laboratory standardization, (iii) biologically representative, and (iv) cost effective, are of significant value to the scientific community.

Our previous analysis of EZ Metric predictive performance revealed that the weighting scheme for the varying zebrafish responses used here has a beneficial influence on the performance of predictive models (Liu et al. 2013); thus, despite the subjective nature of the weighting scheme used to rank the observed biological effects, we feel confident that the weighted EZ Metric is a better predictor than can be achieved using purely additive approach for assessing the sub-lethal endpoints in the assay. The weighted rankings used here may evolve as our understanding of embryonic zebrafish toxicity broadens, but in such an event the EZ Metric score can quickly and easily be recalculated. Combined metrics conducted in vivo that include not only mortality but morphological, developmental, and behavioral endpoints can increase the understanding gained in first-pass screening assays and provide data necessary to improve the development of predictive models. The methods used for the clustering and classification analyses of the NBI dataset are general and may be applied and tested as more data become available; however, as the volume of data and the diversity of the nanoparticles increase, it will be necessary to develop more continuous and mechanistic features that characterize the surface chemistry of the nanoparticles. Models should evolve to incorporate conditional NP features, such as dissolution and zeta potential in a given experimental medium, for building classification models that can predict toxic potential for developing vertebrates.

## Conclusions

Our findings are similar to other studies in embryonic zebrafish which have also suggested that the toxicity of nanomaterials often differs from the toxicity of the core constituents alone (Griffitt et al. 2009; King Heiden 2007; Powers et al. 2010). Yet information gained through these types of rapid assays will need to be considered concomitantly with results in other model systems in order to support applicable risk assessments. Additional high-throughput assays representative of real-world biological diversity should be developed in conjunction with cohesive informatics frameworks, so that the data are sharable and methods and materials are replicable. Our results further suggest that the consideration should be given to the way in which we anticipate regulating nanomaterials. Risk assessments based on nanoparticle core composition alone may be applicable for simple nanoparticles such as metal oxides; however, complex engineered nanoparticle risk assessments may require consideration of their surface constituents, and potentially other conditional physicochemical features like dissolution and zeta potential in a given environment, that are not currently considered.

## Electronic supplementary material

Supplementary material 1 (DOCX 34 kb)
