# Supplementary material for: Comparative hazard analysis and toxicological modeling of diverse nanomaterials using the embryonic zebrafish (EZ) metric of toxicity
Source: J Nanopart Res. 2015 Jun 4;17(6):250. doi: 10.1007/s11051-015-3051-0 (PMC4454819; doi:10.1007/s11051-015-3051-0)
Supplement: Supplementary file 1 — Supplementary material 1 (DOCX 34 kb) [file 11051_2015_3051_MOESM1_ESM.docx]

**Supplementary Information**

**Manuscript: “**Comparative hazard analysis and toxicological modeling of diverse nanomaterials using the embryonic zebrafish (EZ) metric of toxicity**”.**

**Authors:** Bryan Harper, Dennis Thomas, Satish Chikkagoudar, Nathan Baker, Kaizhi Tang, Alejandro Heredia-Langner, Roberto Lins and Stacey Harper.

**Online Resource 1:** Detailed information on nanoparticles included in the study.

| **Publication / Figure Legend Title** | **Core Composition** | **Surface Chemistry** | **Manufacturer** | **Manufacturer #** |
| --- | --- | --- | --- | --- |
| Gold-MEE (2nm) | gold | 2-(2-mercaptoethoxy)ethanol | Jim Hutchison; University of Oregon | N/A |
| Gold-TMAT (0.8nm) | gold | N,N,N-trimethylammoniumethanethiol | Jim Hutchison; University of Oregon | N/A |
| Gold-MES (0.8 nm) | gold | 2-mercaptoethanesulfonate | Jim Hutchison; University of Oregon | N/A |
| Gold-MEE (0.8nm) | gold | 2-(2-mercaptoethoxy)ethanol | Jim Hutchison; University of Oregon | N/A |
| Gold-TMAT (2nm)-ultrapure | gold | N,N,N-trimethylammoniumethanethiol | Jim Hutchison; University of Oregon | N/A |
| Gold-TMAT (2nm)-ultrapure | gold | N,N,N-trimethylammoniumethanethiol | Jim Hutchison; University of Oregon | N/A |
| Gold-TMAT (2nm)-pure | gold | N,N,N-trimethylammoniumethanethiol | Jim Hutchison; University of Oregon | N/A |
| Gold-TMAT (2nm)-as synthesized | gold | N,N,N-trimethylammoniumethanethiol | Jim Hutchison; University of Oregon | N/A |
| Gold-MES (2nm)-ultrapure | gold | 2-mercaptoethanesulfonate | Jim Hutchison; University of Oregon | N/A |
| Gold-MES (2nm)-ultrapure | gold | 2-mercaptoethanesulfonate | Jim Hutchison; University of Oregon | N/A |
| Gold-MES (2nm)-pure | gold | 2-mercaptoethanesulfonate | Jim Hutchison; University of Oregon | N/A |
| Gold-TMAT (2nm)-as synthesized | gold | N,N,N-trimethylammoniumethanethiol | Jim Hutchison; University of Oregon | N/A |
| Gold-TMAT (10nm) | gold | N,N,N-trimethylammoniumethanethiol | Jim Hutchison; University of Oregon | N/A |
| Gold-MHA (10nm) | gold | 6-mercaptohexanoic acid | Jim Hutchison; University of Oregon | N/A |
| Gold-MEEE (0.8nm) | gold | 2,2,2-[mercaptoethoxy(ethoxy)]ethanol | Jim Hutchison; University of Oregon | N/A |
| Gold-MEEE (2nm) | gold | 2,2,2-[mercaptoethoxy(ethoxy)]ethanol | Jim Hutchison; University of Oregon | N/A |
| Gold-MEEE (10nm) | gold | 2,2,2-[mercaptoethoxy(ethoxy)]ethanol | Jim Hutchison; University of Oregon | N/A |
| Gold-MEPA (2nm) | gold | 2-mercaptoethylphosphonic acid | Jim Hutchison; University of Oregon | N/A |
| G3 PAMAM dendrimer - amine | 1,4-diaminobutane STARBURST® PAMAM | amine | Dendritic Nanotechnologies Inc. | DNT-104 |
| G4 PAMAM dendrimer - amine | 1,4-diaminobutane STARBURST® PAMAM | amine | Dendritic Nanotechnologies Inc. | DNT-105 |
| G5 PAMAM dendrimer - amine | 1,4-diaminobutane STARBURST® PAMAM | amine | Dendritic Nanotechnologies Inc. | DNT-106 |
| G6 PAMAM dendrimer - amine | 1,4-diaminobutane STARBURST® PAMAM | amine | Dendritic Nanotechnologies Inc. | DNT-107 |
| G6 PAMAM dendrimer - succinamic acid | 1,4-diaminobutane STARBURST® PAMAM | succinamic acid | Dendritic Nanotechnologies Inc. | DNT-108 |
| G6 PAMAM dendrimer - amidoethanol | 1,4-diaminobutane STARBURST® PAMAM | amidoethanol | Dendritic Nanotechnologies Inc. | DNT-109 |
| Aluminium Oxide (25nm) | aluminium oxide | aluminum oxide | Sigma-Aldrich | 642991 |
| Titanium Dioxide (30nm) | titanium(IV) oxide | titaniun doxide | Sigma-Aldrich | 634662 |
| Zirconium Oxide (50nm) | zirconium(IV) oxide | zirconium oxide | Sigma-Aldrich | 544760 |
| Cerium Oxide (13nm) | cerium(IV) oxide | cerium oxide | Sigma-Aldrich | 544841 |
| Gadolinium Oxide (25nm) | gadolinium(III) oxide | gadolinium oxide | Sigma-Aldrich | 637335 |
| Dysprosium Oxide (25nm) | dysprosium(III) oxide | dysprosium oxide | Sigma-Aldrich | 639664 |
| Yttrium Oxide (25nm) | yttrium(III) oxide | yttrium oxide | Sigma-Aldrich | 641901 |
| Holmium Oxide (25nm) | holmium(III) oxide | holmium oxide | Sigma-Aldrich | 641863 |
| Samarium Oxide (25nm) | samarium(III) oxide | samarium oxide | Sigma-Aldrich | 637319 |
| Silicon Dioxide / Alumina (25nm) | alumina-doped silicon dioxide | alumina doped silicon dioxide | Sigma-Aldrich | 701491 |
| Erbium Oxide (25nm) | erbium(III) oxide | erbium oxide | Sigma-Aldrich | 637343 |
| Polystyrene FluoSphere (20nm) - carboxylated | polystyrene | carboxyl | Invitrogen/Molecular Probes | F8787 |
| Polystyrene FluoSphere (20nm) - sulfonated | polystyrene | sulfate | Invitrogen/Molecular Probes | F8845 |
| Polystyrene FluoSphere (20nm) - aldehyde-sulfate | polystyrene | aldehyde-sulfate | Invitrogen/Molecular Probes | F8760 |
| Gold - phosphatidylcholine (7nm) | gold | phosphatidylcholine | Scott Reed; Portland State University | N/A |
| Gold - phosphatidylcholine (7nm) | gold | phosphatidylcholine | Scott Reed; Portland State University | N/A |
| Gold - phosphatidylcholine (22nm) | gold | phosphatidylcholine | Scott Reed; Portland State University | N/A |
| Gold - phosphatidylcholine (14nm) | gold | phosphatidylcholine | Scott Reed; Portland State University | N/A |
| Gold - phosphatidylcholine (14nm) | gold | phosphatidylcholine | Scott Reed; Portland State University | N/A |
| Nanocrystaline Cellulose - carboxylated | cellulose | carboxyl | John Simonsen; Oregon State University | N/A |
| Nanocrystaline Cellulose - sulfonated | cellulose | sulfate | John Simonsen; Oregon State University | N/A |
| Zinc Oxide - oleic acid (62nm) | zinc oxide | oleic acid | Andreas Stonas; Voxtel | N/A |
| Zinc Oxide - oleic acid (26nm) | zinc oxide | oleic acid | Andreas Stonas; Voxtel | N/A |
| Zinc Oxide (62nm) | zinc oxide | zinc oxide | Sigma-Aldrich | 544906 |
| Zinc Oxide (26nm) | zinc oxide | zinc oxide | Andreas Stonas; Voxtel | N/A |
| Zinc Oxide - octanoic acid acid (62nm) | zinc oxide | octanoic acid | Andreas Stonas; Voxtel | N/A |
| Zinc Oxide - octanoic acid acid (26nm) | zinc oxide | octanoic acid | Andreas Stonas; Voxtel | N/A |
| Zinc Oxide - para-nitrobenzoic acid (62nm) | zinc oxide | para-nitrobenzoic acid | Andreas Stonas; Voxtel | N/A |
| Zinc Oxide - para-nitrobenzoic acid (26nm) | zinc oxide | para-nitrobenzoic acid | Andreas Stonas; Voxtel | N/A |
| Zinc Oxide - cyclohexane carboxilic acid (62nm) | zinc oxide | cyclohexane carboxylic acid | Andreas Stonas; Voxtel | N/A |
| Zinc Oxide - cyclohexane carboxilic acid (26nm) | zinc oxide | cyclohexane carboxylic acid | Andreas Stonas; Voxtel | N/A |
| Zinc Oxide - benzoic acid (62nm) | zinc oxide | benzoic acid | Andreas Stonas; Voxtel | N/A |
| Zinc Oxide - benzoic acid (26nm) | zinc oxide | benzoic acid | Andreas Stonas; Voxtel | N/A |
| Silicon Dioxide - FITC (54nm) | silica dioxide | fluorescein isothiocyanate | Alex Punnoose; Boise State University | N/A |
| Silver - citrate (10nm) | silver | citrate | Nanocomposix Inc. | CTH1148 |
| Silver/Gold - phosphate (21nm) | silver coated gold | phosphate | Nanocomposix Inc. | CTH1085 |
| Silver/Gold - phosphate (33nm) | silver coated gold | phosphate | Nanocomposix Inc. | CTH1088 |
| Silver/Gold - phosphate (41nm) | silver coated gold | phosphate | Nanocomposix Inc. | CTH1083 |
| Silver/Gold - phosphate (53nm) | silver coated gold | phosphate | Nanocomposix Inc. | CTH1092 |
| Silver/Gold - phosphate (61nm) | silver coated gold | phosphate | Nanocomposix Inc. | CTH1082 |
| Silver/Gold - phosphate (68nm) | silver coated gold | phosphate | Nanocomposix Inc. | CTH1108 |
| Silver/Gold - phosphate (70nm) | silver coated gold | phosphate | Nanocomposix Inc. | CTH1039 |
| Silver/Gold - phosphate (92nm) | silver coated gold | phosphate | Nanocomposix Inc. | CTH1112 |
| Silver/Gold - phosphate (101nm) | silver coated gold | phosphate | Nanocomposix Inc. | CTH1084 |
| Silver/Gold - phosphate (122nm) | silver coated gold | phosphate | Nanocomposix Inc. | CTH1066 |
| Zinc Oxide (5nm) | zinc oxide | zinc oxide | Alex Punnoose; Boise State University | N/A |
| Iron Oxide (110nm) | iron oxide | iron oxide | Alex Punnoose; Boise State University | N/A |
| Zinc Oxide (4nm) | zinc oxide | zinc oxide | Alex Punnoose; Boise State University | N/A |
| Lead Sulfide - monothiol, unoxidized (3nm) | lead sulfide | 3-mercaptopropanesulfonic acid, sodium salt | Ian Moody; University of Oregon | N/A |
| Lead Sulfide - monothiol, oxidized (3nm) | lead sulfide | 3-mercaptopropanesulfonic acid, sodium salt | Ian Moody; University of Oregon | N/A |
| Lead Sulfide - dithiol, unoxidized (3nm) | lead sulfide | 2,3-dimercaptopropanesulfonic acid, sodium salt | Ian Moody; University of Oregon | N/A |
| Lead Sulfide - dithiol, oxidized (3nm) | lead sulfide | 2,3-dimercaptopropanesulfonic acid, sodium salt | Ian Moody; University of Oregon | N/A |
| Zinc Oxide (15nm) | zinc oxide | zinc oxide | Alex Punnoose; Boise State University | N/A |
| Zinc Oxide (34nm) | zinc oxide | zinc oxide | Alex Punnoose; Boise State University | N/A |
| Zinc Oxide (5nm) | zinc oxide | zinc oxide | Alex Punnoose; Boise State University | N/A |
| Zinc Oxide (10nm) | zinc oxide | zinc oxide | Alex Punnoose; Boise State University | N/A |
| Zinc Oxide (5nm) | zinc oxide | zinc oxide | Alex Punnoose; Boise State University | N/A |

**Online Resource 2:** Estimated nanomaterial concentrations eliciting 0.1 weighted EZ Metric score used to determine hazard ranking of lower toxicity materials.

| **Material** | **EZM EC_10_** |
| --- | --- |
| Silicon Dioxide - FITC (54nm) | 0.001 |
| Zinc Oxide - benzoic acid (62nm) | 0.001 |
| Nanocrystaline Cellulose - sulfonated | 0.001 |
| Zinc Oxide (10nm) | 0.002 |
| Iron Oxide (110nm) | 0.002 |
| Zinc Oxide (34nm) | 0.002 |
| Zinc Oxide - benzoic acid (62nm) | 0.002 |
| Zinc Oxide - oleic acid (26nm) | 0.003 |
| Nanocrystaline Cellulose - carboxylated | 0.003 |
| Lead Sulfide - dithiol, unoxidized (3nm) | 0.003 |
| Zinc Oxide (5nm) | 0.004 |
| Zinc Oxide (62nm) | 0.004 |
| Zinc Oxide - octanoic acid acid (26nm) | 0.004 |
| Zinc Oxide (26nm) | 0.004 |
| Zinc Oxide - octanoic acid acid (62nm) | 0.004 |
| Zinc Oxide (5nm) | 0.005 |
| Cerium Oxide (13nm) | 0.006 |
| Zinc Oxide (5nm) | 0.011 |
| Zinc Oxide - cyclohexane carboxilic acid (62nm) | 0.015 |
| Zinc Oxide (15nm) | 0.022 |
| Yttrium Oxide (25nm) | 0.050 |
| Zinc Oxide - oleic acid (62nm) | 0.591 |
| Zinc Oxide - para-nitrobenzoic acid (62nm) | 0.601 |
| Polystyrene Fluorosphere (20nm) - sulfonated | 0.849 |
| Polystyrene Fluorosphere (20nm) - aldehyde-sulfate | 0.864 |
| G6 PAMAM dendrimer - amidoethanol | 2.071 |
| Gold-MES (2nm)-ultrapure | 2.406 |
| Zirconium Oxide (50nm) | 7.244 |
| Silicon Dioxide / Alumina (25nm) | 7.674 |
| Gold-TMAT (10nm) | 10.904 |
| Lead Sulfide - dithiol, oxidized (3nm) | 19.555 |
| Gold-MES (2nm)-ultrapure | 20.249 |
| Titanium Dioxide (30nm) | 25.119 |
| Gold-MEPA (2nm) | 50.000 |
| Gold-MES (2nm)-pure | 55.208 |
| Polystyrene Fluorosphere (20nm) - carboxylated | 58.715 |
| Zinc Oxide (4nm) | 60.954 |
| Gold-MES (0.8 nm) | 92.045 |
| Zinc Oxide - cyclohexane carboxilic acid (26nm) | 176.807 |
| G6 PAMAM dendrimer - succinamic acid | 201.837 |

**
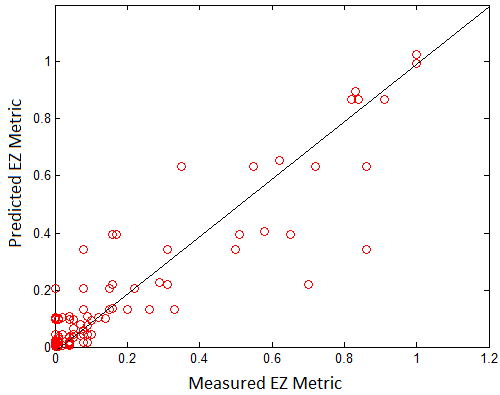
**

**Online Resource 3:** Plot of predicted EZ Metric values obtained with the model in Equation 3 (y-axis) against measured EZ Metric values (x-axis). The black line represents perfect prediction. R-squared value is 0.8811, the adjusted R-squared is 0.8719.
